# Comparison between Short, Medium, and Long Fields of View in Estimating Bicep Femoris Fascicle Length

**DOI:** 10.3390/muscles3020014

**Published:** 2024-05-24

**Authors:** Nicholas J. Ripley, Paul Comfort, John McMahon

**Affiliations:** 1 School of Health and Society, University of Salford, Salford M5 4WT, UK; 2 School of Medical and Health Sciences, Edith Cowan University, Joondalup 6027, Australia; 3 Hawkin Dynamics, Westbrook, ME 04092, USA

**Keywords:** hamstring, fascicle length estimation, ultrasound, single image

## Abstract

Measuring the bicep femoris long head fascicle length via the use of diagnostic ultrasound has become common practice within elite sport, using single images of between 4 and 6 cm. No study to date has compared single image estimations in terms of the varying fields of view (i.e., 4, 6, and 10 cm). Therefore, the aim of this study was to determine whether differences occur when estimating the bicep femoris long head fascicle length using short (4 cm), medium (6 cm), and long (10 cm) fields of view across three estimation equations. A total of 36 male athletes (age: 23.8 ± 3.8 years, body mass: 83.7 ± 14.0 kg, height: 1.81 ± 0.06 m) had three ultrasound images of the bicep femoris long head collected on a single occasion with the fascicle length estimated. A significant main effect was observed (*p* < 0.001) with moderate–very large differences (*p* < 0.078, *d* = 0.91–4.01). The smallest fields of view resulted in the greatest fascicle length. There were significant moderate–large associations between the fields of view (*p* < 0.001, r = 0.542–0.892). Unacceptable limits of agreement were observed, and the developed correction equations remained unacceptable. The partial measure equation is the most accurate whilst using the 10 cm fields of view, while the basic trigonometry equation had the lowest variability between fields of view and the smallest differences between fields of view; hence, this equation may be more appropriate when a <6 cm field of view is the only field of view available.

## 1. Introduction

The bicep femoris long head (BFLH) has multiple roles in both injury prevention and athletic performance [1,2], functioning as a hip extensor and knee flexor [3,4]. The fascicle length (FL) of the BFLH has been reported to influence the muscle’s force–velocity and force–length characteristics [5]. An increased FL through the addition of in-series sarcomeres, which results in a rightward shift of the force–velocity and force–length curves, could contribute to the relationship between the absolute BFLH FL and an elevated risk of hamstring strain injury (HSI) [6,7]. It has been reported that possessing a BFLH FL of <10.56 cm increases the risk of sustaining a HSI 4.1-fold in professional soccer players [6]. Therefore, measuring the BFLH FL via the use of diagnostic ultrasound has become common practice within elite sports [5,6,8]. 

Technology availability is a current limiting factor within ultrasound assessment, with typical probe lengths ranging between 4 and 6 cm [6,7,9,10,11,12]. Therefore, it is impossible to measure the entire length of the BFLH FL from a single image [13], as the observed fascicles generally exceed the probes’ field of view (FOV). As the whole fascicle is generally not in view, the common practice is to utilize tangible architectural measurements and trigonometry to estimate the BFLH FL. Several methods of FL estimation have been utilized; the authors of the present study have investigated the three most used equations previously [14]. The criterion method of estimation is the most used equation in practice, proposed by Blazevich et al. [15] and Kellis et al. [10], which includes measuring the aponeurosis angle (AA) (the curvature of the aponeurosis in relation to the horizontal plane), the pennation angle (PA), and the muscle thickness (MT) (Equation (1)). A secondary method was originally proposed for assessment of the vastus lateralis by Guilhem and colleagues [16], where a partial measure of the viewable fascicle is made followed by utilization of trigonometry to estimate the smallest portion not within the FOV (Equation (2)); this method was used more recently to estimate the BFLH FL [11,13,17]. The third equation, typically used for the symmetrical pennate muscle (vastus lateralis, triceps brachii), which does not consider the AA or any partial measure (Equation (3)), has also been used [14,18]. 

Two methods of ultrasound image acquisition exist within the literature, a single image capture and extended FOV, with extended FOV methods developed to image the entire muscle or entire fascicle of interest [13]. Franchi et al. [13] observed an overestimation when using the extended FOV methods in comparison to the partial measure estimation methods. It should be noted that all methods of single image extrapolation overestimate the BFLH FL in comparison with all extended FOV methods [11,13], whereby the entire fascicle is imaged. This would indicate that extended FOV methods are a superior imaging technique and may be closer to the gold standard; however, extended FOV methods are not without their limitations, requiring skilled ultrasonographers and technical algorithms to merge images [13]. The task-specific skills for extended FOV collection, including ultrasonography, and the technical skills (including coding ability) required [13] do limit the usability of the extended FOV method in elite sports. Time is a crucial component for elite training environments, with sport scientists being under constant pressure with strict time constraints especially within team-sport environments, where a large number of athletes require assessment; this will undoubtedly impact the method selected for muscle architecture capture [14]. Therefore, it could be suggested that single image capture, which is quicker to collect and analyse could be more useable. Researchers have previously demonstrated that all methods of BFLH FL estimation are highly reliable when using both short (<6 cm) and long FOV (10 cm) and can be used to routinely estimate the BFLH FL [6,7,13,14]. 

Recently, it has been identified that when using the three methods of estimation with a 10 cm FOV, there is a significant, albeit trivial, difference between Equations (1) and (3), with very large to nearly perfect relationships between equations; however, due to the unacceptable limits of agreement (LOAs), comparisons between methods should be avoided [14]. Despite these differences, it was highlighted that practitioners could use each of the extrapolation methods to identify meaningful changes in the BFLH muscle architecture [14]. However, no study to date has compared single image estimations between varying FOVs (i.e., 4, 6, and 10 cm), and as differences have already been observed at the largest FOV, it is expected these differences would be consistent. The primary purpose of this study was to compare the BFLH FL estimations between 4, 6, and 10 cm and determine whether any meaningful relationship exists between FOVs. It was hypothesized that there would be significant and meaningful differences in the single image estimations between the FOVs, with the larger FOV enabling a greater accuracy of estimated measures of FL. It was further hypothesized that there would be meaningful associations between FOVs, due to similar tangible measures being identified.

## 2. Materials and Methods

A total of 36 male team sport athletes, identified through convenience sampling (age 23.8 ± 3.8 years, body mass 83.7 ± 14.0 kg, height 1.81 ± 0.06 m) with no history of lower-limb injury or inflammatory conditions within the previous 12 months, had three images of the BFLH captured on the self-identified dominant limb. All participants were also asked to refrain from any exercise 24 h prior to each testing session. The researcher collected and digitized all images collected across both sessions, demonstrating good between-session reliability using the same methods as previously detailed [14]. Written informed consent and the results of a health questionnaire were obtained from all participants prior to testing. The study was approved by the institutional ethics committee (HSR1718-040) and conformed to the principles of the Declaration of Helsinki.

Initially, participants lay in relaxed prone position with full knee and hip extension on a plinth. The scanning site for all images was determined as the halfway point between the ischial tuberosity and the lateral epicondyle, along the line of the BFLH. The images were captured while participants lay relaxed in a prone position. The images were collected along the longitudinal line of the muscle belly utilizing a 2D B-mode ultrasound (MyLab 70 XVision, Esaote, Genoa, Italy) with a 7.5 MHz 10 cm linear array probe, with a depth resolution of 67 mm, an XView of 3, and a density of 48%. To collect the ultrasound images, a layer of conductive gel was placed across the linear array probe, which was then placed over the scanning site and aligned longitudinally to the BFLH, perpendicular to the skin. During the acquisition of the ultrasound images, care was taken to ensure minimal pressure was applied to the skin, as the pressure can distort images, leading to temporarily elongated muscle fascicles [7]. The assessor manipulated the orientation of the probe slightly if the superficial and intermediate aponeuroses were not parallel. 

**Figure 1:**
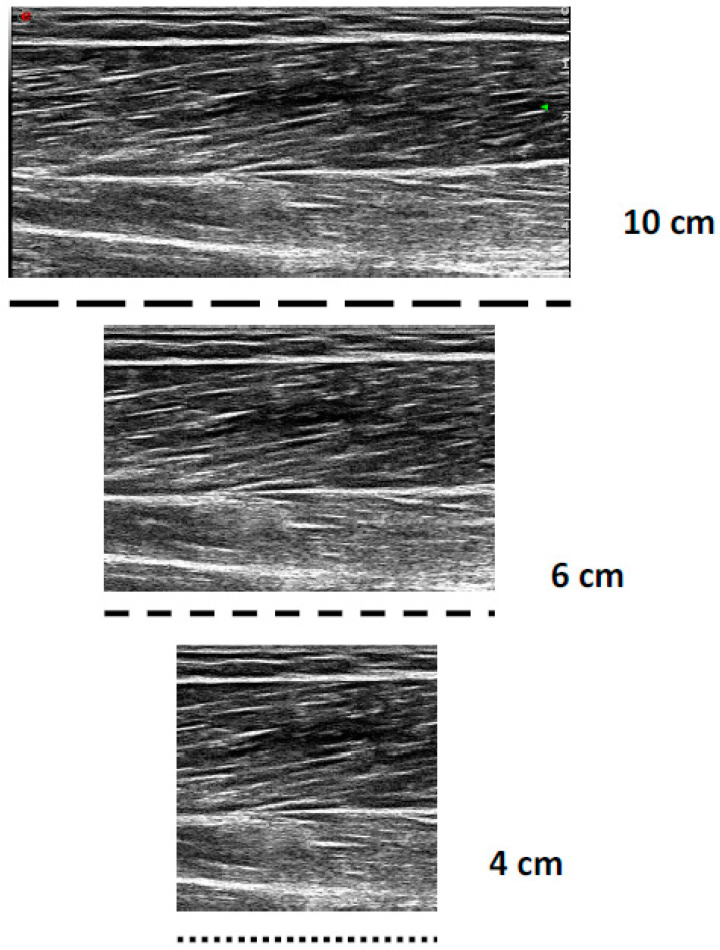
Example differences in short (4 cm), medium (6 cm), and long (10 cm) fields of view when imaging the bicep femoris long head.

All the sonograms were analysed offline with Image J version 1.52 software (Wayne Rasband National Institute of Health, Bethesda, MD, USA). All the images were calibrated to the known length of the FOV (10 cm); then, for the 4 and 6 cm digitization, all images were cropped by 6 and 4 cm, 50% from the distal and proximal portions, respectively, to maintain an image of the muscle belly (Figure 1). For each image (4, 6, and 10 cm), a fascicle of interest was identified, where the *MT*, *PA*, *AA*, and observed *FL* were measured three times within each image, to enable complete *FL* estimation. The 4 and 6 cm FOV were chosen, as these shorter FOVs are the most frequently used within the literature [6,7,9,10,11,12]. Three trigonometric linear equations were utilised within the present study:

*FL* = *SIN* (*AA* + 90 *deg*) × *MT*/*SIN* (180 *deg* − (*AA* + 180 *deg* − *PA*)) (1)

Criterion method of fascicle length estimation.

*FL* = *MT*/(*SIN*(*PA*)) (2)

Fascicle length estimation using basic trigonometry.

*FL* = *L* + (*h* ÷ *SIN*(*β*)) (3)

Fascicle length estimation partial measure equation, where *L* is the observable fascicle length, *h* is the perpendicular distance between the superficial aponeurosis and the fascicles visible end point, and *β* is the angle between the fascicle and the superficial aponeurosis.

Statistical analyses were performed using Jamovi (Jamovi project (2018) Computer Software, retrieved from https://www.jamovi.org, accessed on 12 February 2019). A custom Microsoft Excel spreadsheet was also utilised [19]. Statistical significance was set at *p* < 0.05 for all tests. Normality for all variables was confirmed using a Shapiro–Wilks test, and the data were found to be non-normally distributed (*p* < 0.05). Within-session reliability between the three collected images was assessed via a series of two-way mixed effects intraclass correlation coefficients (ICCs), 95% confidence intervals (CIs), and coefficient of variation (CV). Minimum acceptable absolute reliability was confirmed using a CV < 10% [20]. The ICC values were interpreted based on the lower-bound 95% CI as (<0.49) poor, (0.50–0.74) moderate, (0.75–0.89) good, and (>0.90) excellent [21]. A series of non-parametric repeated measures analysis of variance (Friedman’s) were conducted to determine whether there were significant differences in the FL values between the different FOVs. Durbin–Conover pairwise comparisons were made with Bonferroni correction to adjust for familywise error [22]. Non-parametric Cohen’s *d* effect sizes and 95% Cis, using the formula described by Fritz et al. [23] (*d* = 2r/(1 − r^2^)), were calculated to determine the magnitude of the differences. Cohen’s *d* ES were interpreted as trivial (<0.19), small (0.20–0.59), moderate (0.60–1.19), large (1.20–1.99), and very large (≥2.0) [24].

Spearman’s correlation coefficients, with Bonferroni correction to adjust for familywise error, were used to determine the association between the FOV measures. The correlations were interpreted using the scale described by Hopkins [24]: trivial (<0.10), small (0.10–0.29), moderate (0.30–0.49), large (0.50–0.69), very large (0.7–0.89), nearly perfect (0.9–0.99), and perfect (1.00). The mean of the difference (bias) was expressed in absolute measures, as a percentage, and as a ratio (FOV1/FOV2). The 95% LOA (LOA: mean of the difference ± 1.96 standard deviations) and 95% CI were calculated between FOV measures using the methods described by Bland and Altman [25]. The potential for hetero- or homoscedastic spread was assessed visually using the Bland and Altman plots. Unacceptable LOA were determined a priori as a bias greater than ±5% using the methods described by Bland and Altman [25].

## 3. Results

**Figure 2:**
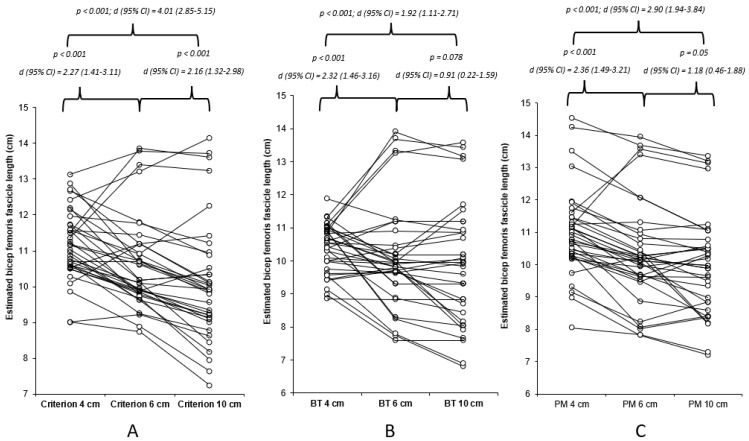
Individual and mean differences (Durbin–Conover pairwise and Cohen’s *d* effect sizes) between 4, 6, and 10 cm FOV for each of the estimation equations utilised within the present study. (**A**) Criterion equation, (**B**) basic trigonometry (BT), and (**C**) partial measure (PM) equation.

**Figure A1:**
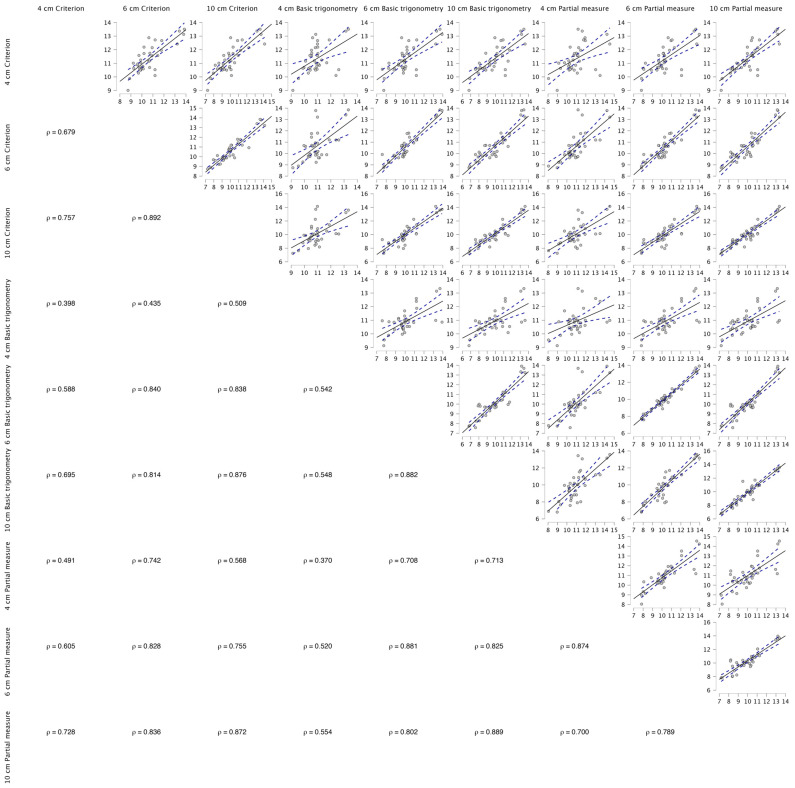
Spearman’s correlations between the fields of view and the three linear equations used within the present study.

The data were found to be non-normally distributed (*p* < 0.05), hence the use of a non-parametric statistical approach, which determined very high and nearly perfect acceptable within-session reliability for all methods of estimation for the 4, 6, and 10 cm FOV methods (Table 1). The results of Freidman’s test revealed a significant mains effect (*p* < 0.001), with the results of the Durbin–Conover pairwise comparisons highlighting significant moderate to very large differences between the FOVs for all estimation methods (Figure 2). The Durbin–Conover pairwise comparisons between estimation equations can found in the Appendix A. Between the images (4 cm, 6 cm, and 10 cm), significant (*p* < 0.001) large to very large associations were observed, with 46–79% of the estimated FL using the criterion equation being able to explain each measure, 29–78% of the estimated FL using the basic trigonometry equation being able to explain each measure, and 49–76% of the estimated FL using the basic trigonometry equation being able to explain each measure (Figure A1). 

**Figure 3:**
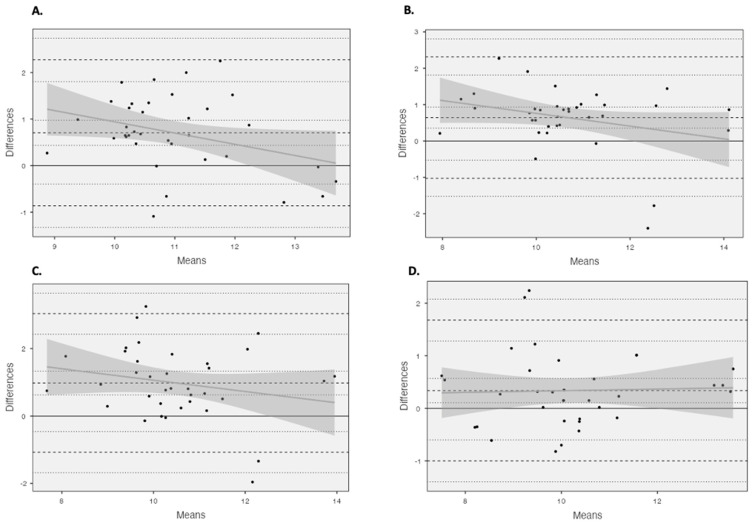
Bland and Altman plots illustrating mean differences and LOA (dashed line) between (**A**) 4 cm and 6 cm using the criterion equation and (**B**) 4 cm and 6 cm using the partial measure equation, as well as (**C**) 4 cm and 10 cm using the partial measure equation and (**D**) 6 cm and 10 cm using the partial measure equation.

Unacceptable LOAs (Table 2) (>5%) were observed for all comparisons. Using Bland and Altman plots (Figure 3), four comparisons were revealed to possess homoscedastic data, 4 cm vs. 6 cm using the criterion equation and all comparisons made using the partial measure equation. The homoscedasticity in addition to large to very large relationships between equations across the fields of view (criterion: r > 0.679, basic trigonometry r > 0.542, partial measure r > 0.700) enabled the development of correction equations (Figure A1). 

The developed correction equations were compared against the original measures, and they were able to correct values to a very minimal mean bias; however, the LOA remained unacceptable (>5%) (Table A1).

## 4. Discussion

The results of this study demonstrated significant and meaningful differences between all FOVs for each of the estimation equations identified, with the largest differences identified between 4 and 10 cm for all the estimation equations. Furthermore, there was an increase in the range of the estimated BFLH FL with increasing FOV for the criterion and basic trigonometry equations (Table 1 and Figure 2), whereas, for the partial measure equation there was a decrease in the BFLH FL range. This is only partially in agreement with the hypothesis of the present study, as the greater ranges observed with the increased FOV for the criterion and basic trigonometry equations could suggest a decreased accuracy because the same tangible measures were taken from all images. However, this is not the case with the partial measure equation, as the greater FOV permits a greater visible FL, which is in agreement with our hypothesis, indicating the greater FOV reduces the degree of estimation and potential error associated with this equation. It is unadvisable to compare the estimated BFLH FL measurements between different FOVs, with unacceptable LOAs (>5%) found for all three estimation equations (Table 2), despite large to very large associations observed between the different FOVs. Correction equations were developed with four of the comparisons, as the data were observed to be homoscedastic (Table A1); however, the ability to correct the original data from the small FOV to match the larger FOV was limited, with an unacceptable LOA (Table A1).

The greater reliability and homoscedastic data observed with partial measure equations could be expected, due to the reduced error, especially when using the larger FOV. When using the larger FOV (i.e., 10 cm), a greater degree of the observed fascicle can be measured, thus reducing the error within the measurement; this is observed with the reduced range of estimated values in addition to the lower measurement variability and relative reliability. This could offer a potential explanation for the consistent overestimation when using a shorter FOV [11,13]. As both the criterion and basic trigonometry equations use the same tangible measurements from all images, the larger FOV could be increasing the variability of these measures due to more information being permitted by the larger single image. Therefore, when increasing the range of the estimated FLs from 4 cm through to 10 cm, although the variability and relative reliability was improved with the larger FOV, the error or bias also could be increased with the larger FOV. This has an impact on method selection, as when practitioners only have access to FOV < 6 cm, it might be prudent to utilize the basic trigonometry equation, as the range of estimated measures is reduced, with low variability and high relative reliability; however, this equation has the lowest magnitude of difference between all FOVs (Figure 1). However, if practitioners have access to a larger FOV (>6 cm), it would be recommended that the partial measure equation be utilised due to the reduced degree of estimation required. Differences were also identified between the estimation equations used; the results can be found in the Appendix A. This is consistent with what has been observed previously at the largest FOV used in the present study [14]. The reliability of all measures is consistent with what has been reported previously across different FOVs and using varying equations [6,7,9,10,11,12], with typically good–excellent relative reliability reported. However, it is worth noting that the interpretation of the relative reliability within the present study has used a more robust method, via considering the lower bound 95% CI, rather than the point estimate used by previous authors [6,7,9,10,11,12]. 

The FOV used to assess the BFLH muscle architecture appears to be a crucial factor when using extrapolation methods to estimate the BFLH FL. Freitas et al. [17] used methods consistent with those in the present study, whereby they observed two single image FOVs, 3 vs. 6 cm, utilising the partial measure estimation equation to calculate the FL. Although no comparative statistical analyses were performed on the differences between the calculated FLs, the 6 cm FOV estimated FLs were lower than the 3 cm comparison, 99.9 mm ± 15.7 and 120.3 mm ± 25.0 for the 6 and 3 cm FOVs, respectively. This is consistent with the results of the present study, whereby the FL measurements achieved using a larger FOV (10 cm) were smaller than those from both the 4 and 6 cm FOVs (Figure 2), to moderate and very large magnitude. The results of the present study are consistent with the conclusions by Franchi and colleagues [13], who noted that the accuracy is dependent upon the length of the visible fascicle, with a shorter FOV not permitting a large proportion of the FL to be visually measured. These differences could have meaningful implications on injury risk assessment within athletes, as when practitioners are using shorter FOV, they need to be aware that they will likely be reporting over-inflated FL measurements.

All Bland and Altman comparisons did not reach acceptable LOAs (<5%), with heteroscedastic data presenting both under- and overestimations, suggesting that the accuracy of these estimation methods could be subject-specific, with no fixed systematic error as per previous findings [11,13]. However, four comparisons presented homoscedastic data, 4 cm vs. 6 cm using the criterion equation and all measures using the partial measure equation, presenting a more fixed systematic error, with an FL overestimation when using the shorter FOV. As these measures presented homoscedastic, correction equations were developed to see whether it would be suitable and appropriate to compare the results from different FOVs. The developed correction equations were able to correct to a minimal mean bias; however, the correction was limited with unacceptable LOAs (>5%). The most appropriate correction was provided when comparing the 6 cm and 10 cm FOVs using the partial measure equation, with the lowest unacceptable LOA (<13%), with the mean bias being identified below the previously established standard error of the measurement values identified for the 10 cm FOV [14], which could be erroneous. This could be related to the fact that the accuracy of the extrapolation is dependent upon the length of the visible fascicle, with a 4 cm FOV being limited to the length of the visible fascicle. However, the LOA for the 6 cm and 10 cm FOVs using the partial measure equation were still unacceptable (>5%), although they were the best of those observed for the homoscedastic data.

Extended FOV methodologies, which aim to image the entire muscle in an attempt to attain an entire visible fascicle, have been established as potentially more accurate methods of BFLH FL assessment, with single image estimations, such as the ones used within the present study, overestimating the BFLH FL and underestimating the PA [11,13]. A potential explanation for these differences includes the poor identification of the superficial aponeurosis trajectory, which could be explained by the fact that both studies compared extended FOVs to single images, using only small FOV ultrasound probes between 5 and 6 cm and not a larger FOV such as the 10 cm probe used within the present study. This could indicate that extended FOV methods are a superior imaging technique [13]; however, several limitations are also present when using extended FOV methods, which makes them less practical in an elite sport setting. Extended FOV methods require skilled ultrasonographers and technical algorithms to merge images. The time required to collect and analyse extended FOV images does limit the usability of the extended FOV method in elite sports, as the time required will undoubtedly increase for both the practitioner and athlete. Time is a crucial component for elite training environments, especially team-sport environments, where many athletes could require testing on multiple occasions (start of pre-season, end of pre-season, mid-season, end of season), with sport scientists being under strict time constraints, which can impact the method selection. Therefore, it could be suggested that single image capture, which is quicker to collect and analyse could be more useable. It is also argued that extended FOV methods are still not considered the “gold standard” for the assessment of the BFLH FL [13,26]. Research should be carried out to determine a “gold standard” method, with particular interest in the third dimension of the muscle using three-dimensional ultrasound—potentially minimizing the impact of the fascicle curvature; however, this adds an extra layer of complexity, which may still be limited in elite team sport environments.

The present study is not without its limitations; firstly, it was only a male team-sport cohort, meaning the findings of the present study may have limited relevance to female athletes, even though both male and female athletes both frequently sustain HSIs. Therefore, future research should look to include female athletes and non-athletic populations to determine whether there any differences in the accuracy of assessments. Moreover, the different FOVs taken for the present study were all based on the same image using the larger FOV (10 cm) and were not taken using different ultrasound devices. If different ultrasound devices were used, there is the potential that the differences observed could inflated due to the varying image quality and imaging physics involved with ultrasonography. 

## 5. Conclusions

As the availability of ultrasound devices increases with affordable wireless devices emerging, the need for practitioners to understand the potential limitations of various methodological considerations when utilising ultrasound is crucial. The present study highlighted that there are significant small to large differences between all FOVs regardless of the estimation equation utilised. Moreover, the largest differences were observed between the smallest and largest FOV (4 vs. 10 cm). The partial measure equation is likely to be the most accurate, especially at a larger FOV, as the accuracy extrapolation is dependent upon the length of the visible fascicle [13], which is undoubtedly dependent on the FOV. However, as the FOV decreases, the accuracy of the extrapolation decreases, which could explain the greater range in values for the 4 cm FOV. However, the basic trigonometry equation had the lowest variability between FOVs and the smallest magnitude differences between FOVs, which means this equation could be more appropriate when only a small FOV is available (<6 cm). If practitioners were looking to compare between normative data or between FOVs, the present study highlights that comparisons made with original data should be avoided due to unacceptable LOAs and heteroscedastic data. Despite the fact that four comparisons presenting homoscedastic data enabled the development of correction equations, corrections and comparisons should be made with caution due to the unacceptable LOA. In conclusion, practitioners should look to use the most appropriate method for them, with a partial measure equation being more appropriate for larger single image FOV (>6 cm); whereas, if using a short single image FOV (<6 cm) due to equipment and time availability, the basic trigonometry equation could be more appropriate due to the reduced variability. However, if a practitioner has time for research or working with a single athlete, it would be recommended to spend the time developing the skills needed for extended FOV measures.

## Figures and Tables

**Table 1:** Median and standard deviation estimated bicep femoris fascicle lengths for 6 and 10 cm field of views and for each estimation method.

	4 cm Field of View			6 cm Field of View			10 cm Field of View		
Equation	Cr	BT	PM	Cr	BT	PM	Cr	BT	PM
Median (cm)	10.85	11.10	10.86	9.93	10.19	10.19	9.94	9.90	9.97
SD	0.88	1.04	1.28	0.77	0.87	0.79	0.87	0.88	0.76
Range (cm)	4.50	4.20	6.49	5.11	6.33	6.13	6.90	6.78	6.16
Min–Max (cm)	9.01–13.51	9.13–13.33	8.05–14.54	8.74–13.85	7.58–13.91	7.82–13.95	7.25–14.15	6.80–13.58	7.20–13.60
CV (%)	3.25	2.86	3.84	1.66	1.56	1.79	0.98	0.84	1.01
95% CI	3.07–3.44	2.70–3.02	3.62–4.06	1.57–1.76	1.47–1.65	1.68–1.89	0.92–1.04	0.79–0.89	0.96–1.07
ICC	0.84	0.90	0.91	0.87	0.97	0.97	0.87	0.94	0.98
95% CI	0.75–0.94	0.83–0.96	0.85–0.98	0.79–0.95	0.95–0.99	0.93–0.98	0.76–0.94	0.88–0.97	0.97–0.99

Cr = criterion, BT = basic trigonometry, PM = partial measure, SD = standard deviation, CV = coefficient of variation, CI = confidence interval, ICC = intraclass correlation coefficient.

**Table 2:** Bias and limits of agreement between the estimated measures of bicep femoris fascicle length between the 4, 6, and 10 cm fields of view.

			Limits of Agreement			Ratio (SD)
			Lower	to	Upper	
Criterion 4 cm vs. 6 cm	Bias	0.705	−0.863	-	2.273	1.07 (0.08)
	95% CI	0.435 to 0.976	−1.330 to −0.396	-	1.806 to 2.740	
	Percent Bias (%)	6.98	−8.54	-	22.50	
Criterion 4 cm vs. 10 cm	Bias	1.246	−0.689	-	3.181	1.14 (0.11)
	95% CI	0.912 to 1.580	−1.265 to −0.112	-	2.605 to 3.758	
	Percent Bias (%)	12.61	−6.97	-	32.19	
Criterion 6 cm vs. 10 cm	Bias	0.541	−0.768	-	1.851	1.06 (0.07)
	95% CI	0.315 to 0.767	−1.158 to −0.378	-	1.461 to 2.241	
	Percent Bias (%)	5.48	−7.77	-	18.73	
Basic trigonometry 4 cm vs. 6 cm	Bias	0.819	−1.442	-	3.081	1.10 (0.12)
	95% CI	0.429 to 1.21	−2.115 to −0.768	-	2.407 to 3.754	
	Percent Bias (%)	7.71	−13.57	-	28.99	
Basic trigonometry 4 cm vs. 10 cm	Bias	1.041	−1.67	-	3.753	1.13 (0.16)
	95% CI	0.573 to 1.509	−2.477 to −0.863	-	2.945 to 4.560	
	Percent Bias (%)	10.32	−16.56	-	37.21	
Basic trigonometry 6 cm vs. 10 cm	Bias	0.222	−1.216	-	1.66	1.03 (0.03)
	95% CI	−0.026 to 0.470	−1.216 to −0.788	-	1.121 to 2.088	
	Percent Bias (%)	2.20	−12.06	-	16.46	
Partial measure 4 cm vs. 6 cm	Bias	0.642	−1.026	-	2.311	1.08 (0.09)
	95% CI	0.354 to 0.930	−1.523 to −0.529	-	1.814 to 2.807	
	Percent Bias (%)	6.20	−9.92	-	22.33	
Partial measure 4 cm vs. 10 cm	Bias	0.981	−1.073	-	3.036	1.12 (0.11)
	95% CI	0.627 to 1.336	−1.685 to −0.461	-	2.424 to 3.647	
	Percent Bias (%)	9.80	−10.72	-	30.33	
Partial measure 6 cm vs. 10 cm	Bias	0.339	−1.000	-	1.679	1.04 (0.08)
	95% CI	0.108 to 0.570	−1.399 to −0.601	-	1.280 to 2.078	
	Percent Bias (%)	3.39	−9.99	-	16.78	

SD = standard deviation, CI = confidence interval.

## Data Availability

The data presented in this study are available on request from the corresponding author (accurately indicate status).

## Appendix A

**Table A1:** Bias and limits of agreement between the estimated measures of bicep femoris fascicle length between the 4, 6, and 10 cm fields of view and corrected measures.

				Limits of Agreement			Ratio (SD)
Ct 6 cm vs. Corrected (4 cm)	6 cm FL = 0.9743 × Alternative − 0.415	Bias	0.4	−1.642	-	2.441	1.11 (0.090)
		95% CI	0.047 to 0.752	−2.250 to −1.034	-	1.833 to 3.049	
		Percent Bias (%)	3.96	−16.25	-	24.16	
PM 6 cm vs. Corrected (4 cm)	6 cm FL = 0.9988 × Alternative − 0.6851	Bias	0.002	1.785	-	1.786	1.00 (0.09)
		95% CI	0.108 to 0.570	−1.399 to −0.601	-	1.280 to 2.078	
		Percent Bias (%)	0.02	17.25	-	17.26	
PM 10 cm vs. Corrected (4 cm)	10 cm FL = 0.8903 × Alternative + 0.1738	Bias	1.91	−2.045	-	2.047	1.00 (0.10)
		95% CI	0.108 to 0.570	−2.655 to −1.436	-	1.438 to 2.656	
		Percent Bias (%)	19.08	−20.43	-	20.45	
PM 10 cm vs. Corrected (6 cm)	10 cm FL = 0.8902 × Alternative + 0.7971	Bias	0.09	−1.296	-	1.297	1.00 (0.07)
		95% CI	0.108 to 0.570	−1.399 to −0.601	-	1.280 to 2.078	
		Percent Bias (%)	0.90	−12.95	-	12.96	
